# How does human motor cortex regulate vocal pitch in singers?

**DOI:** 10.1098/rsos.172208

**Published:** 2018-08-15

**Authors:** Michel Belyk, Yune S. Lee, Steven Brown

**Affiliations:** 1 Bloorview Research Institute, Holland Bloorview Kids Rehabilitation Hospital, Toronto, Ontario, Canada; 2 Department of Speech and Hearing Sciences and Center for Brain Injury, The Ohio State University, Columbus, OH, USA; 3 Department of Psychology, Neuroscience & Behaviour, McMaster University, Hamilton, Ontario, Canada

**Keywords:** voice, pitch, larynx, motor cortex, functional magnetic resonance imaging, speech

## Abstract

Vocal pitch is used as an important communicative device by humans, as found in the melodic dimension of both speech and song. Vocal pitch is determined by the degree of tension in the vocal folds of the larynx, which itself is influenced by complex and nonlinear interactions among the laryngeal muscles. The relationship between these muscles and vocal pitch has been described by a mathematical model in the form of a set of ‘control rules’. We searched for the biological implementation of these control rules in the larynx motor cortex of the human brain. We scanned choral singers with functional magnetic resonance imaging as they produced discrete pitches at four different levels across their vocal range. While the locations of the larynx motor activations varied across singers, the activation peaks for the four pitch levels were highly consistent within each individual singer. This result was corroborated using multi-voxel pattern analysis, which demonstrated an absence of patterned activations differentiating any pairing of pitch levels. The complex and nonlinear relationships between the multiple laryngeal muscles that control vocal pitch may obscure the neural encoding of vocal pitch in the brain.

## 1. Introduction

The modulation of vocal pitch in humans is central to the communication of meaning through both speech prosody and musical melody [1–6]. This includes both the discrete pitch movements found in much of music and the more-continuous pitch transitions that are found in speech and the expression of emotion. Such modulations of pitch are mediated by the brain's control over the muscles of the larynx. Although there is a growing body of research on the vocal motor system of the human brain, relatively little is known about the representation of pitch within this system, not least compared to the well-established spatial representation of frequency in the auditory system.

**Figure 1.**
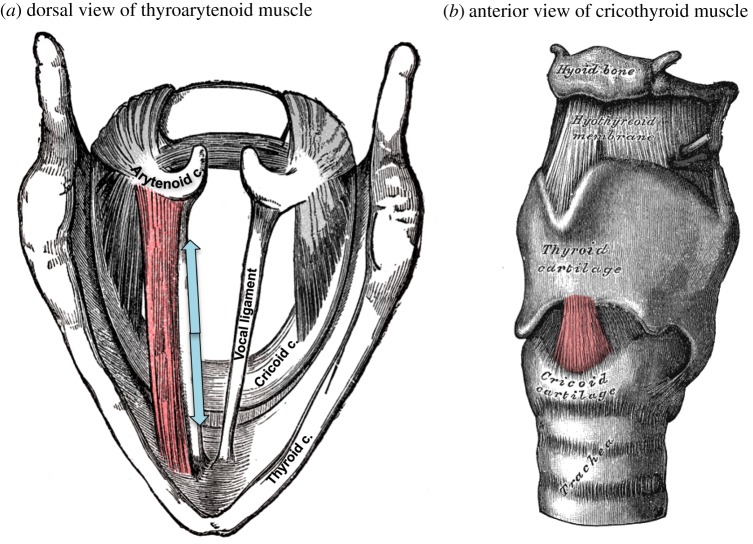
The thyroarytenoid muscle (TA; *a*) and cricothyroid muscle (CT; *b*) are the primary controllers of vocal pitch. The CT rocks the thyroid cartilage forward, thereby stretching the vocal folds and raising vocal pitch. The TA lies within the vocal folds themselves and causes them to become shorter and stiffer, and has a nonlinear influence on vocal pitch. Other laryngeal muscles (depicted in grey) serve to adduct and abduct the vocal folds, effectively turning voicing on or off, or to raise and lower the entire larynx within the airway. These latter muscles have only a minor influence on vocal pitch. Drawings are modified from Gray [8].

Vocalization—also known as phonation or voicing—is produced by the vibration of two fibrous elastic membranes inside the larynx. These vocal folds vibrate passively as air is forced through the vocal tract, where the fundamental frequency (F0) of this vibration is determined by an adjustable set of physical properties of the vocal folds [7]. These physical parameters are controlled primarily by two major intrinsic laryngeal muscles, namely the cricothyroid (CT) and thyroarytenoid (TA) muscles (figure 1). Contraction of the CT muscle stretches and increases the tension of the vocal folds, causing them to vibrate at a higher F0 and raising the pitch of the voice [9–13]. The TA muscle lies within the body of the vocal folds themselves. Contraction of this muscle may either shorten the vocal folds to lower vocal pitch or stiffen them to raise vocal pitch. The net influence of the TA muscle depends strongly on interactions with the CT muscle, the range of frequencies being produced and the amplitude of vocalization [14,15].

The dynamics of vocal pitch have been described by a mathematical model of ‘control rules’ mapping combinations of laryngeal-muscle movements onto their influence over vocal pitch [7]. This model predicts the oscillations of the vocal folds as a function of a low-dimensional set of parameters describing the configuration of the vocal folds, such as their compressional stiffness, length, thickness and depth. In turn, it predicts the configuration of the vocal folds from the degree of contraction of certain laryngeal muscles, most notably the CT and TA muscles. The control rules map combinations of CT and TA contraction onto vocal pitch modulations. How these control rules are implemented in the central nervous system to regulate vocal pitch during speech, song and expressions of emotion is presently unknown.

The lower motor neurons that innervate the intrinsic laryngeal muscles are found in the nucleus ambiguus in the medulla. In rat models of vocalization, this nucleus contains separate somatotopic divisions for the lower motor neurons that innervate the CT and TA muscles, respectively [16]. In monkeys, the cortical larynx area also contains separate representations of the CT, TA and other intrinsic laryngeal muscles [17]. However, the cytoarchitecture and function of the larynx area in humans differ markedly from those in all other apes [17–22], highlighting the need for human research in this area. Several transcranial magnetic stimulation (TMS) studies in humans have observed electromyographic activity in the cricothyroid muscle following magnetic stimulation of the ventral primary motor cortex [23–26]. One study in humans observed considerable spatial separation between two scalp locations that preferentially activated the CT and TA muscles, respectively [27], although another study that recorded from the same muscles observed a single shared cortical locus [28].

Neuroimaging studies using functional magnetic resonance imaging (fMRI) have successfully distinguished between the larynx motor cortex (LMC) and adjacent orofacial somatotopic modules in primary motor cortex (M1) that control movement of the lips and tongue [29–31]. Few studies have attempted to discern how the LMC performs its primary function in controlling vocal pitch. Peck *et al*. [32] had participants phonate at three pitch levels in their vocal range, Howell *et al*. [33] examined the production of rising and falling tones in Mandarin, and Kryshtopava *et al*. [34] examined phonation at both comfortable and high pitch levels, when compared with silent expiration. None of these experiments observed differences in the locations of activations in the LMC as a function of vocal pitch. Possible reasons for the inability of these studies to detect pitch-dependent modulation of the LMC include: (i) the tendency of untrained singers to couple pitch modulation with vertical movements of the larynx that engage a complex of extrinsic laryngeal muscles, which may obscure activity related to the muscles with the strongest influence on vocal pitch, (ii) the insensitivity of standard general linear model (GLM) analyses of fMRI data to distinguish adjacent or overlapping activations, (iii) the nonlinear relationship between the profile of activation of the intrinsic laryngeal muscles (CT and TA) and vocal pitch, and (iv) the neural representations of the various laryngeal muscles in the human brain might be more overlapping than predicted from animal models.

We report an fMRI study in which chorally trained singers vocalized discrete pitches at four different levels within their pitch range. We examined singers who were able to decouple vocal-pitch production from vertical movements of the larynx. In addition to running standard GLM analyses to assess gross brain activation, we extracted the coordinates of the peak activations from each participant to test for small-scale differences in the location of LMC activations at each pitch level. Finally, we applied multi-voxel pattern analysis (MVPA) to leverage the sensitivity of machine-learning methods to spatial patterns that may not be observable with standard univariate analyses. We hypothesized that if the CT and TA muscles are controlled by separate cortical loci, vocal-pitch levels that differentially engage these two muscles would preferentially activate different cortical subregions or produce different patterns of activation within the LMC.

## 2. Methods

### 2.1. Participants

Twelve participants (seven males, five females), with a mean age of 27.0 years (ranging from 16 to 48 years), participated in the study after giving their informed consent. Each individual was without neurological or psychiatric illness. Participants were all fluent English speakers (11 native speakers of English, one of Japanese). One male participant was left-handed. All participants were chorally trained singers, with 4–18 years of choral singing experience (mean = 9.5, s.d. = 4.7).

### 2.2. Procedure

During a training session on a day prior to fMRI scanning, we collected vocal recordings of each participant in order to obtain their habitual speaking pitch and effective vocal range. We had each participant sing a stable and comfortable pitch using the neutral vowel schwa, then sweep down to the lowest pitch that they could comfortably produce without altering the quality of their voice (e.g. without producing creaky voice or vocal fry). The same procedure was repeated with an upward sweep to estimate the highest pitch that each participant could comfortably produce. Each participant's lowest reliably produced pitch became their ‘low’ pitch. Three additional pitch levels were selected by determining the musical interval that is a perfect fifth above the preceding pitch. This is equivalent to taking a 3 : 2 frequency ratio. For example, if the low pitch of a participant was 100 Hz, then the three remaining pitches would be 150 Hz, 225 Hz and 337.5 Hz, respectively. We chose to use an increment of a perfect fifth in order to cover a large part of the vocal range without forcing participants to transition into the falsetto register. The three pitch levels above ‘low’ are hereafter referred to as ‘comfort’, ‘mid’ and ‘high’, respectively. The second pitch level tended to approximate each participant's comfortable or habitual pitch, and the final pitch was near the upper limit of the chest-voice range for most participants.

We instructed the participants to perform phonation as soft hums during the first half of a relaxed breath phrase, hence roughly the first 3 s of a 6 s breath phrase, followed by a gentle and controlled nasal inspiration. All phonation was done nasally, rather than orally, in order to match the vocal-tract configuration of the quiet breathing that constituted the baseline condition of ‘rest’ (see below). During training, we synthesized voice-like complex waves at each pitch level in order to provide participants with an auditory template of the sound to be produced. Visual inspection of a participant's thyroid prominence confirmed that the larynx did not move vertically within the airway during the production of any pitch level. The training session was recorded using Adobe Audition CC (v. 9.2.1.19), which provided an online display of the recorded waveform. This display was provided as biofeedback to train participants to produce each pitch level at an equal and soft amplitude. Training continued until participants were able to reliably produce each pitch level as instructed with no observable head movement.

Vocalization tasks have been observed to produce activation artefacts in fMRI experiments [35]. Tissue movement near the limit of the MRI's field of view can induce changes in the magnetic field gradients that encode spatial locations within the scanner. Of principal concern are articulatory movements of the tongue and vertical movements of the larynx. Our task design mitigated concerns over tongue movement by having participants vocalize with the same neutral vowel (schwa) across conditions to keep the position of the tongue constant. Our participant-recruitment strategy mitigated concern over vertical laryngeal movements. Untrained singers tend to recruit the extrinsic laryngeal muscles to raise or lower the larynx as a whole when they modulate vocal pitch, despite the modest influence of these muscles on F0 [13,36], but trained singers can suppress these movements [37–39]. We verified that all participants were able to sing across the stimulus range without moving the larynx vertically within the airway.

In the scanner, the tasks were performed according to a block design, alternating between 20 s of phonation and 20 s of rest. Given that a breath phrase for production was roughly 6 s, participants typically made three such breath phrases of the same pitch during each 20 s task epoch. During each task epoch, a visual text-cue indicating which of the four pitch levels the participant should sing was projected from an LCD projector onto a screen mounted at the head of the MRI table with an angled mirror on the head coil that reflected text from the screen into the participant's field of view. At the beginning of each phonation block, an auditory cue played over MR-compatible headphones provided a participant-specific template for the pitch level to be phonated during that block. During the rest periods, the word ‘Rest’ was projected onto the screen. Participants were instructed to keep their eyes on a crosshair in the centre of their field of view at all times. All stimuli were presented using the Presentation^®^ software (v. 14.4, www.neurobs.com). Each participant completed four runs of 16 blocks. Every run contained four blocks of each pitch level, occurring in pseudorandom order.

### 2.3. Magnetic resonance imaging

Magnetic resonance images were acquired with a General Electric Achieva 3-Tesla MRI at the Imaging Research Centre at St. Joseph's Hospital in Hamilton, Ontario. The participant's head was firmly secured using foam pillows. Earplugs were used to help block out scanner noise. The imaging parameters were 2500 ms TR, 35 ms TE, 90^o^ flip angle, 30 slices, 3 mm slice thickness, 0 mm gap, 2.25 × 2.25 mm in plane resolution, 64 × 64 matrix and 192 mm field of view. A total of 260 volumes were acquired. Four dummy volumes were discarded, leaving 256 volumes over 10′40″ of scan time, corresponding with 16 alternations between 20 s epochs of task and 20 s epochs of rest. T1-weighted anatomical images were collected for image registration with the parameters 7.47 ms TR, 2.1 ms TE, 164 slices, 2 mm slice thickness, 0.4688 × 0.4688 mm in plane resolution, 512 × 512 matrix and 240 mm field of view.

### 2.4. Image analysis

MRI data were processed with SPM12 (Wellcome Trust Centre for Neuroimaging, London, UK). All images were realigned to the first echoplanar image. Functional runs were co-registered to the T1-weighted images for each individual participant, and spatially normalized into Montreal Neurological Institute (MNI) standard stereotactic space using a transformation matrix generated during tissue class segmentation [40]. No spatial smoothing was performed in order to avoid a loss of effective spatial resolution.

### 2.5. Whole-brain general linear model

Statistical parametric maps were computed in order to contrast phonation versus rest (combining the four pitch levels), in addition to each individual pitch level versus rest. We tested for gross changes in the location of LMC activations as a function of pitch at the group level. The BOLD (blood oxygen level-dependent) response was modelled using boxcar predictors for phonation (with all pitch levels combined) and for the low, comfort, mid and high pitches separately. First-level fixed-effects analyses were conducted for each participant, and parameter estimates were forwarded to a random-effects analysis to assess significance at the level of the group. The group-level map was generated with a cluster-forming threshold of *p* < 0.01 and corrected for multiple comparisons with cluster thresholds computed by permutation test (cluster-wise *p* < 0.05) [41,42] as implemented in the Statistical Non-Parametric Mapping toolbox (SnPM13, http://warwick.ac.uk/snpm, retrieved 1 May 2018).

### 2.6. Centroid analysis

We compared the coordinates of maximal activation among the four pitch levels. Separate statistical maps were computed for each participant at each pitch level. Coordinates of maximal activation within the primary motor cortex were extracted from fixed-effects analyses that were computed separately for each participant. We extracted separate peak-activation coordinates from the fundus and wall of the central sulcus by identifying the activation peaks nearest to these sites.

Linear mixed effects (LME) models were used to test the hypothesis that the coordinates of any pitch level differed from any other pitch level using the lme4 package in R (v. 3.4.1) [43,44]. We constructed LME models to predict coordinate values for activation peaks in either hemisphere from the fixed factors (i) pitch level (low, comfort, mid and high) and (ii) cardinal direction (*x*, *y* and *z*), with a random intercept of participant. If the locations of activation peaks varied by pitch level, we expected a significant interaction between pitch level and one or more of the cardinal directions.

### 2.7. Multivariate pattern classification

After preprocessing, the fMRI time series of all voxels was extracted from the functional images. These raw signals were temporally high-pass filtered with a 300 s cut-off to remove signals unrelated to the neural activity (e.g. linear drift) and were standardized across the four runs to adjust intensity differences among the runs. Intensity vectors were obtained within the range around the peak of HRF (haemodynamic response function) based on the boxcar model of the block design. These vectors were then submitted to the Gaussian Naive Bayes (GNB) linear classifier [45] in the Matlab 2013a statistics toolbox (Mathworks Inc., Natick, MA, USA). Using the whole-brain searchlight analysis [46], we performed four-way classification at every local searchlight sphere, which comprises a centre voxel and its neighbouring voxels within a three-voxel radius. The classifier was initially trained by data in three runs in order to build a model that set the boundary among the neural vectors associated with each of the four pitch conditions. The model was then applied to the data in the one remaining run, in which the accuracy was computed by summing the number of correct classifications of each of the four labels (chance level = 25%). This procedure was repeated four times, such that each combination of the four runs served as a training set (i.e. fourfold cross-validation). The classification accuracy for each searchlight sphere was averaged across the fourfold cross-validation and written in each of the centre voxels in the searchlight output image. The individual searchlight output map was then submitted to random effects analysis after the chance level was adjusted from 0.25 to 0 for a one-sample *t*-test in SPM12. The group-level map was generated with a threshold of *p* (voxel-wise uncorrected) less than 0.001 with corrected cluster size (*p* < 0.05) using the family-wise rate correction method [41,42].

## 3. Results

### 3.1. Whole-brain voxel-wise general linear model

**Figure 2.**
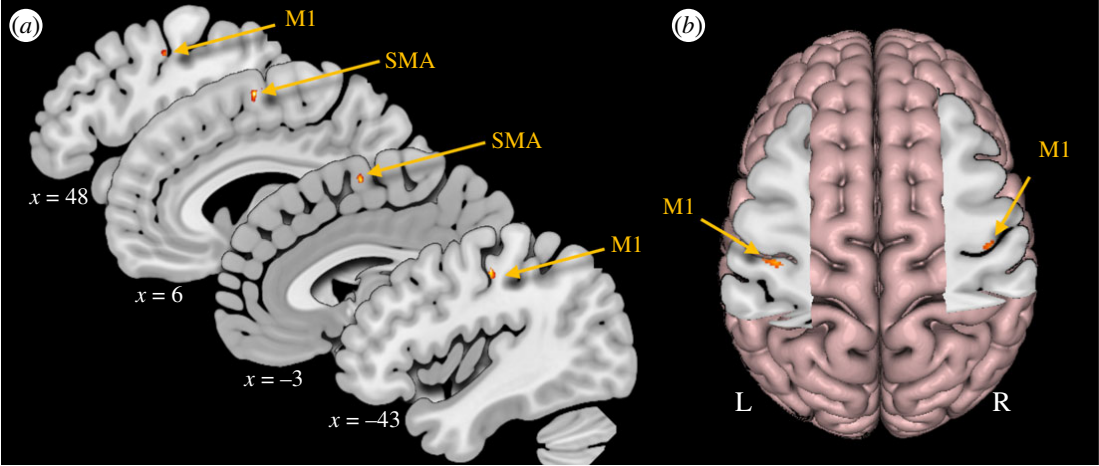
Activations from random-effects analyses for phonation. (*a*) Sagittal slices showing all activations from the contrast phonation versus rest. (*b*) The cut-out shows axial slices at the level of *z* = 45 and 41, in the left and right hemisphere, respectively. M1, primary motor cortex; SMA, supplementary motor area.

Group-level activations are summarized by the contrast of phonation versus rest across pitch levels. This contrast revealed significant activations bilaterally in the primary motor cortex (M1) and supplementary motor area (SMA; table 1 and figure 2).

**Table 1.** MNI coordinates (*x*, *y*, *z*) of activation peaks from significant clusters for the group-level analysis of the contrast of phonation versus rest; *t* indicates the test statistic and *k* indicates the cluster extent in voxels. A cluster-size threshold of *k* < 47 was computed by a permutation test. M1, primary motor cortex; SMA, supplementary motor area.

task	*x*	*y*	*z*	*t*	*k*
M1	−43	−18	46	4.09	126
M1	48	−13	42	3.4	55
SMA	6	−2	63	4.32	176
SMA	2	7	62	3.38	—
SMA	2	−3	70	2.98	—
SMA	−3	−3	61	4.04	99

Group-level analyses for each of the four pitch levels yielded highly overlapping pericentral activations (coordinates are presented in table 2). For the mid- and high-pitch conditions, separate activation clusters were observed along the wall and fundus of the central sulcus, respectively. The SMA and precuneus were also more active during phonation at certain pitch levels compared to rest, presumably due to non-pitch-related experimental demands, such as initiating vocalization and reading the visual text-cues.

**Table 2.** MNI coordinates (*x*, *y*, *z*) of activation peaks from significant clusters for the group-level analysis for individual pitch levels versus rest; *t* indicates the test statistic and *k* indicates the cluster extent in voxels. Cluster-size thresholds of *k* > 39, *k* > 42, *k* > 47 and *k* > 42 were computed by a permutation test for the low-, comfort-, mid- and high-pitch levels, respectively. M1, primary motor cortex; SMA, supplementary motor area.

low	*x*	*y*	*z*	*t*	*k*
M1-wall	−43	−18	44	4.4	88
SMA	6	2	64	5.31	251
SMA	−4	−3	61	4.48	128
SMA	18	2	64	5.07	69

comfort	*x*	*y*	*z*	*t*	*k*
M1-wall	−42	−19	47	3.38	84
M1-wall	48	−14	41	4.25	66
SMA	6	−2	64	3.76	59

middle	*x*	*y*	*z*	*t*	*k*
M1-wall	−47	−17	43	4.64	130
M1-wall	47	−15	40	3.78	131
M1-fundus	40	−16	35	2.56	—
SMA	−7	−4	61	4.59	73
SMA	2	−3	70	3.23	72

high	*x*	*y*	*z*	*t*	*k*
M1-fundus	40	−15	35	3.05	61
M1-wall	−42	−18	46	4.46	174
M1-wall	52	−13	48	2.82	137
M1-wall	47	−14	41	2.77	—
SMA	−5	−2	61	3.09	127
SMA	3	6	63	2.67	—
precuneus	26	−73	25	6.52	72

### 3.2. Individual centroid analysis

In two-thirds of the participants, pericentral activations were observed in the expected range of the LMC at the fundus of the central sulcus in both hemispheres. All participants also exhibited additional activation peaks along the wall of the central sulcus. In most participants, the two peaks occurred within a single cluster spanning both areas, although in a minority of participants these peaks were found in separate non-contiguous clusters.

**Figure 3.**
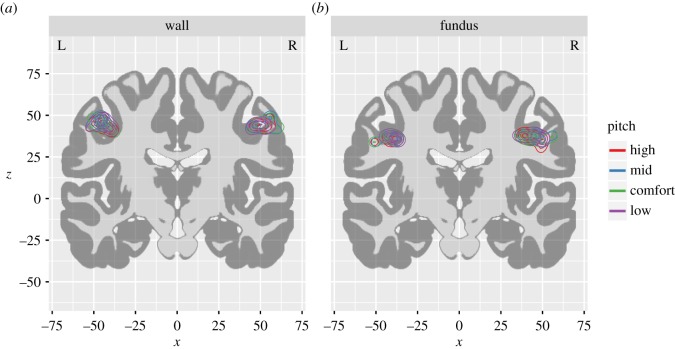
Contour plots of activation peaks by pitch level. The density of activation peaks extracted from individual participants is projected onto a coronal slice (*y* = −14) that intersects both the fundus and wall pericentral clusters. Density distributions for the fundus peaks are displayed in (*a*), and peaks for the wall are displayed in (*b*). Density distributions reflect the local prevalence of activation peaks at each point in stereotaxic space. Each successive ring of the contour plot outlines regions of successively lower density, such that the bulk of the peak activations are observed near the central ring and fewer peaks are observed near the peripheral rings. Within each region-of-interest, the density distributions for the different pitch levels are highly overlapping.

Centroid coordinates varied considerably across individuals, but were highly consistent among pitch levels within each individual (figure 3). In 57% of the hemispheres in which the LMC was detected, we observed identical loci of peak activation for at least two different pitch levels. In the remainder of cases, activation peaks for different pitch levels were found in adjacent voxels. Linear mixed models detected no evidence of differences between centroid locations in either hemisphere for the fundus or along the wall of the central sulcus (all *F*-values < 1, all *p*-values > 0.4). Table 3 lists the mean and standard deviation for the coordinates of peak activation at both sites in both hemispheres. The same results were obtained after excluding the one participant who was left-handed.

**Table 3.** Mean and standard deviation of the MNI coordinates (*x*, *y*, *z*) in the primary motor cortex.

	left				right		
	*x*	*y*	*z*		*x*	*y*	*z*
wall of central sulcus							
high	−44.8 (4.3)	−18.3 (4.2)	44.3 (2.7)	high	50.7 (4.2)	−8 (3.5)	44.3 (2.1)
middle	−46.2 (3.3)	−17.4 (3.5)	45.8 (2.3)	middle	52.7 (5.5)	−7.9 (4.1)	44.6 (3.0)
comfort	−44.1 (5.0)	−18.3 (3.5)	43.7 (3.1)	comfort	52.2 (6.0)	−9.5 (2.0)	44.2 (2.3)
low	−43.5 (4.5)	−18.4 (4.0)	44.1 (2.7)	low	49.6 (4.1)	−10 (4.4)	43.5 (1.5)
fundus of central sulcus							
high	−41.5 (5.2)	−19.8 (3.0)	35.7 (1.5)	high	43.6 (5.2)	−14 (3.9)	37.2 (1.7)
middle	−41.7 (4.6)	−17.7 (4.0)	36.2 (1.5)	middle	45.1 (6.2)	−13.5 (5.6)	37.25 (1.6)
comfort	−42.2 (5.5)	−20 (3.8)	36.2 (1.5)	comfort	44.7 (6.5)	−13 (8.6)	36.7 (2.7)
low	−39.8 (3.3)	−19 (4.5)	36.5 (1.0)	low	44.3 (5.8)	−14.1 (4.6)	37.6 (0.8)

### 3.3. Magnitudes of activation

**Figure 4.**
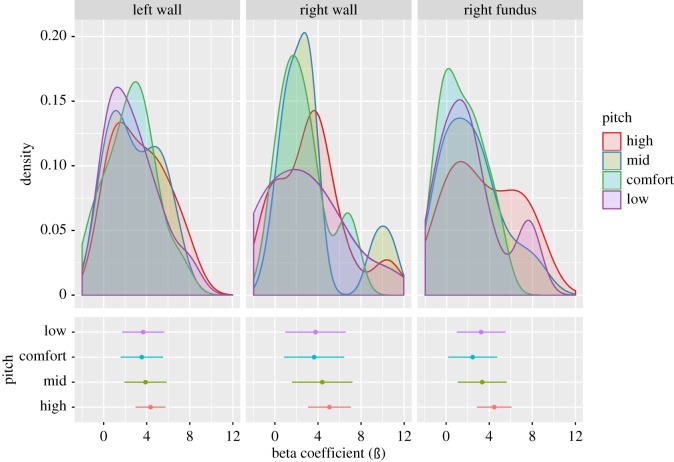
Distribution of activation magnitudes by pitch level. The upper panel plots the distribution of estimate parameters from the fixed-effects analyses for each participant at each pitch level. The lower panel plots mean parameter estimates (ß) and confidence intervals from these distributions. All panels are plotted with common *x*-axes to facilitate comparison.

Beta values extracted from regions-of-interest for the fundus and wall that were identified in the group analysis did not vary as a function of phonated pitch level (figure 4, all *F*-values < 1.1, all *p*-values > 0.3). The same result was obtained after excluding the one participant who was left-handed.

### 3.4. Multivariate pattern classification

Consistent with the individual centroid and magnitude-of-activation analyses, the four-way searchlight analysis did not yield any peak voxels in the LMC. However, secondary findings were observed in the cuneus, precuneus, middle occipital gyrus, posterior cingulate cortex, anterior cingulate cortex, superior frontal gyrus and putamen, as well as in non-LMC areas of the motor cortex associated with representations of the respiratory and articulatory muscles, but not in that part of the primary motor cortex that was identified by the GLM. The same result was obtained after excluding the one participant who was left-handed.

## 4. Discussion

We had participants hum at different pitch levels across their vocal range during fMRI scanning in order to search for the biological implementation of control rules that govern the coordination of the laryngeal muscles that modulate vocal pitch, which is central to the production of both speech and song in humans. Compared to previous fMRI studies that have looked at the vocalization of different pitch levels [32–34], we used trained singers for this study so as to ensure comparable amplitudes of production across different vocal registers, as well as to reduce the tendency of novice singers to raise their larynx with rising vocal pitch, thereby activating the extrinsic laryngeal musculature. The results showed that, while individual participants differed among themselves in the locations of peak activation in the LMC, these locations were similar across pitch levels within all participants. Consistent with this finding, MVPA in the LMC did not distinguish between pitch levels, despite the high sensitivity of this method [46,47]. We observed no evidence that phonated pitches are encoded by either coarse-grained spatial locations or fine-grained spatial patterns in the primary motor cortex.

### 4.1. Activation in the fundus and wall of the central sulcus

We observed two sets of activation peaks within the primary motor cortex. The most prominent peak was observed along the wall of the central sulcus, and a second peak was observed more ventrally and medially at the fundus of the central sulcus. The latter is consistent with the expected location of the LMC from previous neuroimaging studies [29,48–53], although activation spanning both sites have also been reported [31,48,54,55]. In the light of the observation that the magnitudes of activation did not change across pitch levels either in the fundus or along the wall of the central sulcus, it is unlikely that these two sites reflect a separation between the CT and TA muscles.

### 4.2. The encoding of pitch in the larynx motor cortex remains elusive

We report the most direct, sensitive and controlled experiment to date to examine the neural encoding of vocal pitch in the human primary motor cortex using a cohort of trained singers. Peck *et al*. [32] observed a single locus of activation in the LMC across pitch levels, but possible differences were found in other parts of the vocal-motor network, including the inferior frontal gyrus (IFG), cerebellum and putamen. Howell *et al*. [33] likewise observed a single locus in the LMC across pitch levels, but found differences elsewhere, including the cerebellum and anterior insula. Although both of these studies observed differences in a similar region of the cerebellum, Peck *et al*. observed that the cerebellum was associated with producing a high pitch, while Howell *et al*. observed that it was associated with lowering pitch. Kryshtopava *et al*. [34] observed few differences even between vocalization and exhalation, and these were located in auditory association areas and the brainstem, rather than in the primary motor cortex. That the pattern of findings outside of the primary motor cortex is idiosyncratic suggests that they are related to the differing task demands of these experiments, rather than to pitch control *per se*. The LMC is still the most plausible candidate region for vocal pitch control, although the biological implementation of the control rules for vocal pitch remains elusive.

### 4.3. Small-scale organization of small muscles in motor cortex

The elusiveness of the biological implementation of the control rules for vocal pitch may be a symptom of the broader uncertainty in the organization of the primary motor cortex on the spatial scale of small and adjacent muscles. Although the gross separation of M1 into leg, arm and face divisions is uncontroversial [20,56–60], there remains some debate about the degree to which each of these areas can be subdivided into smaller units of muscular anatomy and whether they represent individual muscles or combinations of synergistic muscles that together produce a movement [61]. Smaller units of anatomy appear to have separable but overlapping representations in M1. Several researchers have observed partial overlap between adjacent muscles of the arm and hand using both centroid [62–65] and MVPA-based analyses [66]. This pattern appears to hold even for the distribution of individual upper motor neurons in the primary motor cortex [67]. Similarly, the orofacial division of the primary motor cortex contains separable, but possibly overlapping, representations of at least the larynx, lips, tongue and jaw, as suggested by both fMRI [29,30,50,68] and electrophysiological experiments with neurosurgical patients [18,20,21,57]. Although observable limb movements can be decoded from fMRI data using machine-learning techniques, such as MVPA [69,70], we were unable to decode pitch levels in the LMC, which are the most readily observable outcomes of the actions of the intrinsic laryngeal muscles.

### 4.4. The one-to-many and many-to-one problems in larynx motor control

The difficulty in searching for representations of the CT and TA muscles may be compounded by the integrative role of the human LMC in coordinating respiration with a complex of laryngeal muscles, only some of which have a strong influence on vocal pitch. Likewise, the relationship between the laryngeal muscles and vocal pitch is complex and nonlinear, which is problematic for using vocal pitch as an indirect assessment of these muscles.

#### 4.4.1. One-to-many: the human larynx motor cortex controls a multitude of muscles

The LMC controls not only the CT and TA muscles that are the primary drivers of vocal pitch [18,21], but also muscles that adduct and abduct the vocal folds to cycle between voiced and voiceless sound production [29,71], the extrinsic laryngeal muscles that raise and lower the larynx within the airway [72], as well as the muscles of expiration [52,53]. We have previously predicted that the LMC, in addition to its projections to the larynx motor neurons in the nucleus ambiguus of the brainstem [73,74], may also have novel projections to the respiratory motor neurons of the nucleus retroambiguus to support this broad muscular profile [75]. The diverse set of muscles affected by the LMC may obscure the relationship between this brain region and vocal pitch.

It is also possible that the control of vocal pitch may be distributed between the two cerebral hemispheres, because muscles on either side of the larynx probably receive simultaneous inputs from the right and left LMCs. The larynx is a midline structure, and the two vocal folds operate symmetrically and synchronously as a coordinated pair during normal functioning. The LMC projects to lower motor neurons bilaterally, such that both sides of the larynx receive input from both cerebral hemispheres [73,76,77].

#### 4.4.2. Many-to-one: a multitude of muscles affect the larynx

The problem of motor equivalence may further obscure the relationship between the LMC and vocal pitch. A given pitch level can be reached by multiple configurations of the CT and TA muscles [14,15]. Likewise, factors that exert an external force on the laryngeal frame may affect vocal pitch, such as the engagement of the extrinsic laryngeal muscles that raise or lower the larynx, the position of the tongue and jaw, and the state of the diaphragm during vocalization [37,78]. Hence, although flexibility across physical contexts is one of the hallmarks of M1 [79], the muscles of vocalization are subject to a large degree of nonlinear interaction, both with muscles that are controlled by the LMC and with those that are not. Further studies seeking to address this question may require invasive electromyography recordings of the laryngeal muscles combined with brain imaging, or the greater spatial and temporal resolution of neuro-navigated TMS [26,80].

### 4.5. Limitations

The necessity of indirectly assessing laryngeal motor output through vocal acoustics, rather than by direct observation of muscular contractions or their resulting movements, may obscure the relationship between cortical activity and vocal pitch. A further possibility is that the integrative nature of the LMC—coordinating the actions of the intrinsic laryngeal muscles, extrinsic laryngeal muscles and respiratory muscles—may lead the neural profiles of phonation at different pitch levels to be more similar than different. The degrees of freedom of the movements that modulate vocal pitch are large, and the combinations of movements that lead to a particular pitch outcome on any given trial may not be readily observable within the MRI environment. The spatial resolution of the functional images may have been too coarse to decode meaningful patterns in the LMC.

We recruited experienced singers for their high degree of control over the laryngeal musculature so as to isolate the CT and TA muscles and mitigate vocal tract movement-related imaging artefacts. However, experience-dependent cortical plasticity may have led this population of participants to have enlarged representations of the laryngeal muscles [81,82]. In addition, participants were heterogeneous with respect to sex, handedness and native language, which may have increased inter-individual variability in the LMC. However, even within individual participants, there was little variation in neural activation as a function of vocal pitch.

## 5. Conclusion

The modulation of vocal pitch is critical to human communication processes, including speech and song. Pitch control by the brain is one of the unanswered questions in the neuroscience of human vocal communication. How the brain controls the complex set of rules that coordinate the laryngeal muscles to modulate vocal pitch in speech, song and emotional expression remains elusive. Despite applying the most sensitive statistical tools available, we were unable to observe any relationship between brain activation in the LMC and modulations of vocal pitch. The primary motor cortex remains the most promising candidate for the locus of these control rules, although further methodological developments may be required to advance this line of inquiry.

## Supplementary Material

Peak activations at the fundus of the central sulcus

## Supplementary Material

Peak activations on the wall of the central sulcus

## Supplementary Material

R code to process peak coordinate data

## Data accessibility

Summary data are available in the electronic supplementary material. The full dataset is available from Dryad at: https://datadryad.org/resource/doi:10.5061/dryad.nf3t684 [83].
